# Fragment-based virtual screening discovers potential new *Plasmodium* PI4KIIIβ ligands

**DOI:** 10.1186/s13065-022-00812-2

**Published:** 2022-03-24

**Authors:** Akachukwu Ibezim, Mbanefo S. Madukaife, Sochi C. Osigwe, Nadja Engel, Ramanathan Karuppasamy, Fidele Ntie-Kang

**Affiliations:** 1 Department of Pharmaceutical and Medicinal Chemistry, University of Nigeria, Nsukka, Nigeria; 2 Department of Statistics, University of Nigeria, Nsukka, Nigeria; 3 Department of Oral, Maxillofacial and Plastic Surgery, Rostock University Medical Center, Rostock, Germany; 4 Department of Biotechnology, Vellore Institute of Technology, Vellore, 632014 Tamilnadu India; 5 Department of Chemistry, University of Buea, Buea, Cameroun; 6 Institute of Pharmacy, Martin-Luther University Halle-Wittenberg, Halle, Germany

**Keywords:** *Plasmodium*, Malaria, Type III beta phosphatidylinositol 4-kinase, Fragment-based, Homology modelling, Molecular dynamics

## Abstract

Type III beta phosphatidylinositol 4-kinase (PI4KIIIβ) is the only clinically validated drug target in *Plasmodium* kinases and therefore a critical target in developing novel drugs for malaria. Current PI4KIIIβ inhibitors have solubility and off-target problems. Here we set out to identify new Plasmodium PI4K ligands that could serve as leads for the development of new antimalarial drugs by building a *P*PI4K homology model since there was no available three-dimensional structure of *Pf*PI4K and virtually screened a small library of ~ 22 000 fragments against it. Sixteen compounds from the fragment-based virtual screening (FBVS) were selected based on ≤ − 9.0 kcal/mol binding free energy cut-off value. These were subjected to similarity and sub-structure searching after they had passed PAINS screening and the obtained derivatives showed improved binding affinity for *Pf*PI4K (− 10.00 to − 13.80 kcal/mol). Moreover, binding hypothesis of the top-scoring compound (**31**) was confirmed in a 100 ns molecular dynamics simulation and its binding pose retrieved after the system had converged at about 10 ns into the evolution was described to lay foundation for a rationale chemical-modification to optimize binding to *Pf*PI4K. Overall, compound **31** appears to be a viable starting point for the development of *P*PI4K inhibitors with antimalarial activity.

## Introduction

Malaria is a fatal human parasitic disease caused by five species of *Plasmodium*, with *P. falciparum* as the deadliest species, and was responsible for more than 220 million clinical cases and about half a million deaths in 2018 alone [1–3]. Children under the age of five and pregnant women are most vulnerable and above 90% of the cases occurred in the sub-Saharan African region alone according to the World Health Organization (WHO) 2019 report [4, 5]. There has been progress in controlling the disease through drug therapies, the use of insecticides, and the design of vaccines. However, it has persisted because of a plethora of reasons spanning from non-adherence to prescription by patients, emergence of *Plasmodium* drug-resistant strains to resistance to commonly used insecticides among the mosquito vector population [6–8]. Undoubtedly, the need for an effective antimalarial drug is urgent and many researchers tend to agree that targeting an enzyme that is vital in all *Plasmodium* life cycle will do the job [9]. An example of such a target is type III beta phosphatidylinositol 4-kinase (PI4K) [10, 11]. PI4KIIIβ is a ubiquitous eukaryotic enzyme that plays an essential role in merozoite development through the regulation of intracellular signaling, cytokinesis, and membrane trafficking by catalyzing the conversion of phosphatidylinositol (PI) to phosphatidylinositol-4-phosphate (PI4P) [9–11]. A study by Sternberg and Roepe [12] identified *P*PI4K as an operational enzyme in all *Plasmodium* life-cycle stages and further provided genetic, chemical, and biochemical evidence to show that *P*PI4K is a suitable target for antimalarial drug and its inhibition can cure, prevent and block the transmission of the disease. Paquet and his coworkers discovered a *P*PI4K inhibitor, **MMV390048** that is potent against multiple life cycle stages of the malaria parasite [13]. Even though at the moment **MMV390048** is in Phase IIa human clinical trials as a new malaria therapeutic, this compound is reported to potently exhibit off-target effect which raises the question of safety [11, 14]. Also, many inhibitors of the protein have failed to progress to clinical testing due to poor solubility. Several scientists have virtually screened many small-molecule libraries [15, 16] and chemical series such as the thiazolo-pyrimidinamine series [17], the imidazopyridazine series [18], and the naphthyridine series [19] to discover *P*PI4K inhibitors, further underpinning the relevance of this enzyme in anti-malarial drug search. However, to the best of our knowledge, no study has so far applied a fragment-based screening strategy to discover *P*PI4K ligands. Over the past two decades fragment-based drug discovery has been demonstrated to be one of the most prominent alternatives to high throughput screening utilized in the identification of lead compounds in drug discovery [20, 21]. It begins with finding low fragments or low molecular weight compounds that bind weakly to the target of interest. The fragments that form high quality interactions are maximized to lead compounds with high affinity and selectivity [22].

In the present study, a *P*PI4K homology model was constructed using PI4K of human crystal structure as a template, followed by developing a molecular docking protocol using a series of PI4K inhibitors and non-inhibitors curated from ChEMBL database. This was applied to screen a small library of fragment molecules. Pool of compounds derived from fragment hits were re-docked to investigate their improved binding affinities and molecular dynamics simulation was applied to evaluate binding stability. The result of this study is capable of leading to new *P*PI4K inhibitor templates for designing novel antimalarial drugs.

## Materials and methods

### Homology modelling

Since there is currently no X-ray crystal structure of *Pf*PI4K, a homology model was constructed using its amino acid sequence taken from the NCBI website [23]. During BLAST, the target sequence was used to search RCSB Protein Data Bank to identify the template structure employed in this study (PDB ID: 6GL3) [24]. Modeller (version 9.21) [25] was used to build the homology model and subsequently the selection of the homology model for this study was based on the assessment of the Modeller objective function and Discrete Optimization Protein Energy. PROCHECK [26] and Discovery Studio [27] programs were used to evaluate the quality of the best models.

### Preparation of molecules and virtual screening

The dataset used in this study were retrieved from two compound libraries: the fragments and fragment derived subset were retrieved from ZINC-15 database [28] while PI4K inhibitors and non-inhibitors were obtained from ChEMBL database [29], protonated and energy minimized with OpenBabel software [30] to prepare them for molecular simulation processes.

The homology modeled *P*PI4K structure was prepared for molecular simulation purposes following the protocol previously described by Ibezim et al. [31]. The Protonate 3D module and the Merck Molecular force-field (MMFF94) force field [32] implemented in Molecular Operating Environment (MOE) [33] were respectively used to protonate the amino acid residues (with the pH set at 7.0 ± 2.0) and to minimize the energy of the modeled *P*PI4K structure to a potential energy gradient of 10^–15^ kcal/mol so as to optimally orient the protein atoms to the lowest energy level.

To conduct molecular docking, first the modeled protein was aligned to the template protein–ligand complex (PDB ID: 6GL3) to locate the binding site. Afterwards, a grid box measuring 42 × 22 × 20 points with 0.375 Å point spacing was generated around the identified protein active site and then both the inhibitors and non-inhibitors were docked into the grid using the AutoDock suite as described in our earlier work [34]. An area under curve (AUC) was plotted using R program to determine the capability of the AutoDock program to differentiate between inhibitors and non-inhibitors of PI4K. The validated virtual screening protocol was used thereafter to carry out molecular docking of the study compounds.

An online platform was used to filter the fragment hits against PAINS [35] while MACSS structural keys were used to assess their structural novelty by comparing them to known *P*PI4K inhibitors. Similar and substructures of candidates which passed both assessments (PAINS and novelty check) were retrieved from ZINC-15 database and submitted to docking calculations following the protocol described above.

### Molecular dynamics simulation

The parameter file of ligand was generated by Automated Topology Builder (ATB) [36] whereas the molecular dynamics simulation was carried out by Gromacs ver4.5.5 using GROMOS96 (ffG53a6) force-field [37]. The protein–ligand complex was submerged into solvated cubic box simple point charge (SPC) comprising an explicit water model and sufficient sodium and chloride ions which serve to neutralize the system. Steepest-descent integrator and conjugate gradient algorithm were applied to perform energy minimization. The system was agitated for a period of 100 ns during which solvent molecules and ions were allowed to equilibrate around the solute molecule at 300 k and 1 bar while all the non-hydrogen atoms were subjected to position restraining force.

## Results and discussion

### Construction of *P*PI4K homology model

**Fig. 1:**
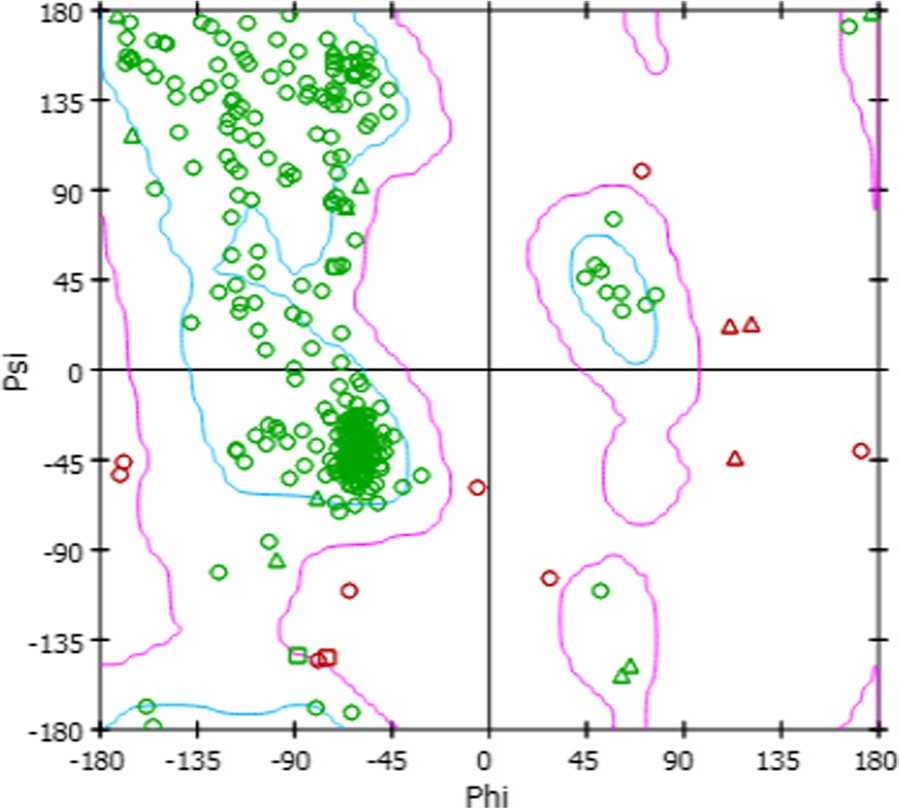
Ramachandran plot for the *Pf*PI4K receptor homology model

*Pf*PI4K sequence with ID: KNG744841.1 was used as query sequence to BLAST (Basic Local Alignment Search Tool) the protein databank through NCBI (National Center for Biotechnology Information) online platform and the algorithm identified human PI4K (Access ID: 6GL3) as the most suitable template even though they share sequence identity of 44.36%. Our decision to go ahead with 6GL3 as the template structure in spite of the relatively low sequence identity was because it has an inhibitor bound within the ATP-site which stretches beyond the ribose pocket to create ATP binding pocket with large conformation in the P-loops. The advantage is that the large binding pocket will allow the accommodation of molecules of varying structural sizes, thereby expanding the possibility of identifying hits of diverse and novel structural motif. The three dimensional structure of PPI4K was constructed using Modeller program from the selected template. After several models were built by the modeller program, the best model was selected based on the DOPE scoring function. PROCHECK identified that 77.0%, 19.70%, 2.60%, and 0.70% of the protein model residues are respectively located in the core, allowed, the general and disallowed region as shown by the Ramachandran plot in Fig. 1. Since the stereo-chemical quality of the homology model was satisfactory, it was then used in this study.

### Evaluation of virtual screening workflow

**Fig. 2:**
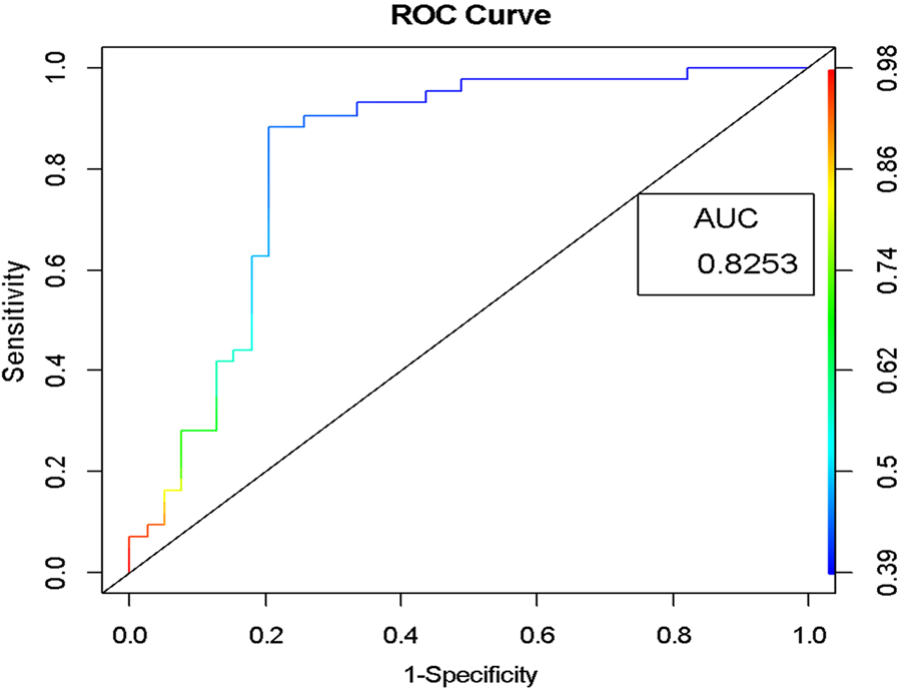
Assessment of the performance of virtual screening protocol: Area Under ROC curve

To ensure reliable results, docking protocols are validated. This is achieved through two broad approaches: in the first approach the capability of docking program to reproduce experimental pose of a co-crystallized ligand is accessed by measuring the root mean square deviation (rmsd method) while in the second approach the correlation between dock score and the enzyme inhibition concentration (IC_50_) of compounds is determined by measuring some statistical metrics such as R^2^, AUC score [38, 39]. Apparently the rmsd method requires a co-crystallized ligand and since that is not available in this study we chose the latter method. Several docking protocols were generated and their abilities to discriminate between PI4K inhibitors and non-inhibitors were evaluated using a dataset curated from the ChEMBL database in which compounds with IC_50_ < 10 µM are grouped as PI4K inhibitors and those with IC_50_ > 10 µM are grouped as non-inhibitors. All the inhibitors curated here were assayed by ADP-Glo Kinase Assay (Promega) method; where the activity of the PI4K is determined by measuring the quantity of adenosine diphosphate (ADP) produced during the kinase reaction [40] whereas other methods such as “in situ PI4K2A kinase assay with COS-7 cells” [41] were also used in screening the non-inhibitors. The ratio of inhibitors to non-inhibitors in the validation set was 1:5 (that is 30 out of 150 molecules). Figure 2 shows the enrichment curve for the acceptable docking protocol and explains that the enrichment is better than random since according to the area under the ROC (AUR), the protocol demonstrated 82.53% overall prediction accuracy. Therefore, it can retrieve potential PI4K inhibitors from a database of diverse molecules in a virtual screening scheme.

### Fragment-based virtual screening

**Fig. 3:**
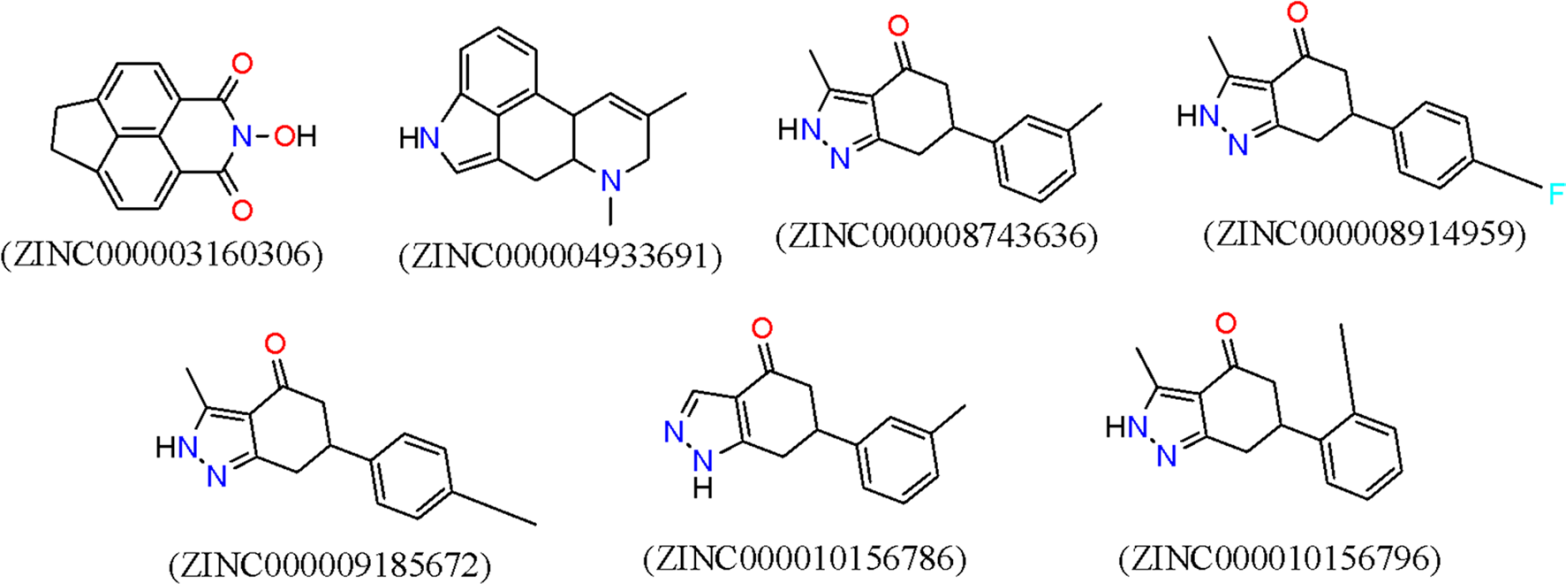
The seven fragments with uncommon structural scaffolds from other known PI4K inhibitors

A subset of 21 844 unique fragments extracted from ZINC database (comprises 727 842 purchasable compounds and a total of over 34 million molecules) was docked towards the binding site of *P*PI4K model using the validated docking protocol. The choice of fragment-based technique is based on two reasons: (1) fragments cover large chemical space which invariably increases the chances of identifying new PI4K inhibitors; and (2) the technique is reported to identify more hits than other virtual screening methods [42]. The binding free energy of the screened fragments spanned from positive values to as low as − 9.31 kcal/mol. Using binding energy ≤ − 9.00 kcal/mol as cut-off, 16 fragments were selected and submitted for pan-assay interference compounds (PAINS) filtration to identify frequent hits which often are promiscuous binders. Two molecules were caught by the filter while the remaining 14 fragments passed. To focus our study on molecules with unusual structural scaffolds different from already known PI4K inhibitors, Tanimoto coefficient calculation using MACCS structural fingerprints at the overlap of 60% to known PI4K ligands curated from the ChEMBL database was performed and this resulted in seven fragments with 2 uncommon scaffolds (Fig. 3). Through visual inspection, the two scaffolds were identified as isoquinoline and indazolone.

To explore ligand space ZINC-15 database, comprising of over 34 million molecules, was explored to retrieve similar and substructures of the seven structurally unique top-scoring fragments. This exercise led to 131 fragment derivatives and results of their docking calculations demonstrated that each of the derivatives exhibited enhanced binding affinities for *P*PI4K over their parent fragments. This is expected given that the derivatives generally possess better frames that suitably accommodate the protein binding pocket (except in the event a bulky substituent group clashes with protein residues) and greater number of moieties capable of engaging into more interactions with binding site residues [43]. There was no clear structure–activity relationship, however, it appears the protein preferred interaction with the derivatives of isoquinoline than indazolone scaffold because the isoquinolines showed comparable greater binding affinity (≤ − 11.00 kcal/mol) (Table 1) than the indazolones (most of them have binding free energies in -10 kcal/mol range). The performance of the isoquinoline series further suggests that the N-2 position does not permit flexible moieties since all the derivatives (compounds **5**, **6**, **10**, **11**, **13**, **14**, **22**) with a flexible group at that position have relatively low scores (≥ − 10.00 kcal/mol). On the other hand, the presence of rigid group(s) and moieties capable of making electrostatic interaction at the N-2 and para positions of the two benzene rings tends to enhance binding with *P*PI4K. That is probably the reason compound **29** has better binding energy than **4** and **7** (Table 1). Of all the derivatives, compound **31** exhibited the highest binding affinity for *P*PI4K (− 13.80 kcal/mol). Results of computed molecular descriptors for **31** (Table 2) suggest it possesses interesting physicochemical profile that could surmount solubility challenge facing known PI4K inhibitors.

**Table 1:**
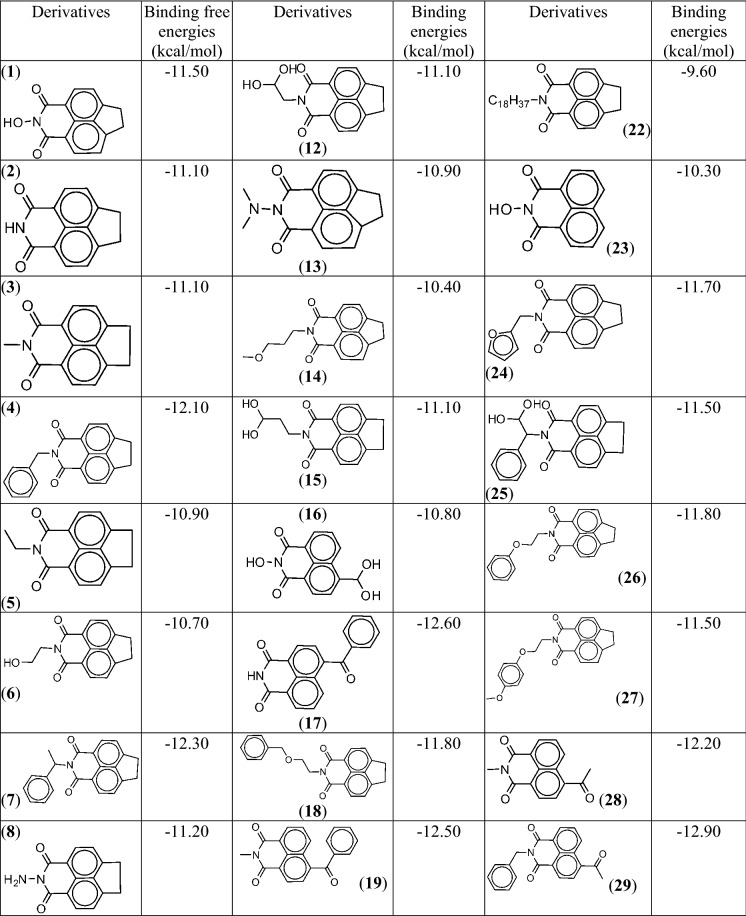

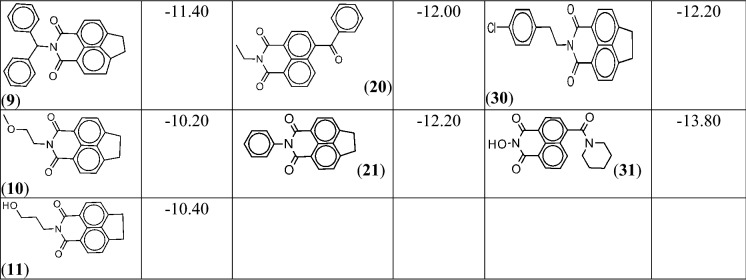
The derivatives of the best performing scaffold and their binding free energies

**Table 2:** Basic physical parameter of compound **31**

Molecular descriptor	Value
MW	324.33
logP	2.45
HBA	6
HBD	1
NRB	2
TPSA	197.84
NHA	24

TPSA, total polar surface area (Å^2^); NRB, number of rotatable bonds; HBD, hydrogen bond acceptor; HBD, hydrogen bond donor; logP lipophilicity; MW, molar weight (Da); NHA, number of heavy atoms

### Molecular dynamics simulation

**Fig. 4:**
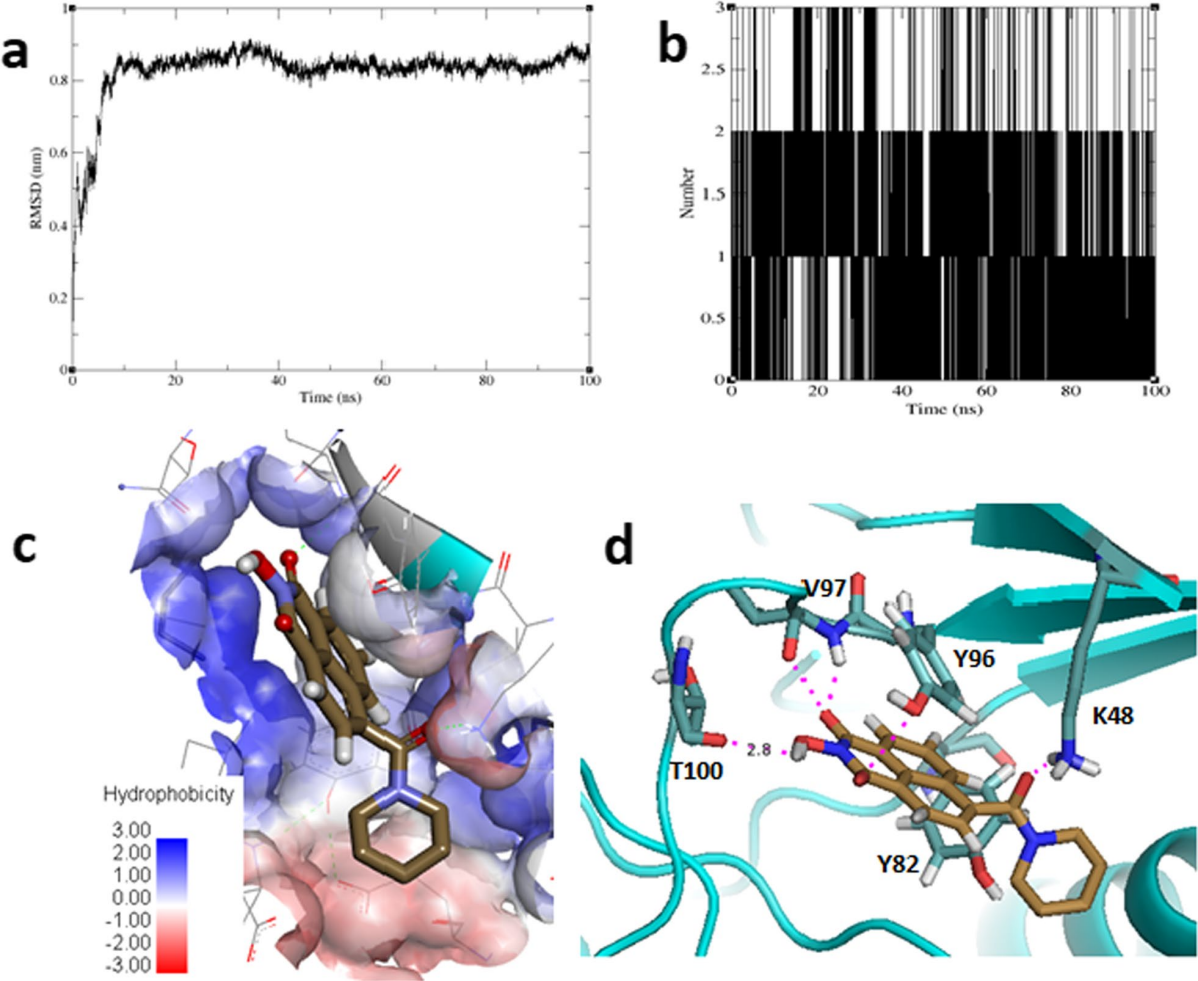
The performance of compound **31** across the 100 ns production phase trajectory. **a** RMSD of the *P*PI4K-compound **31** complex system, **b** Hydrogen bond count formed between *P*PI4K and compound **31**, **c** Hydrophobic surface of PPI4K binding site with overlay of the ligand and **d** Predicted ligand pose at the system convergence. Carbons of the protein residues and the ligand are colored cyan and light brown respectively while hydrogen bonds are shown in magenta colored dotted lines. Discovery Studio and PyMol software were used to generate subfigures 1c and 1d respectively

The presence of a ligand in a protein system causes the system to adjust from its initial state (without the ligand) to new spatial conformation (with the ligand). If the ligand binds with the protein favorably, the system converges (that is; the system reaches the new spatial conformation and remains stable) and vice versa [44, 45]. In MD simulations, rmsd is calculated to determine the spatial difference between the starting structure of the simulation and all succeeding frames. Figure 4a shows that compound **31** had a stable binding interaction with *P*PI4K since the system reached equilibrium shortly before 10 ns and continued to be stable for the rest of the 100 ns it was allowed to evolve. Figure 4b depicts that strong hydrogen bonds were present between the compound and the protein active site residues at each point of the trajectory. In comparison to the ligand pose before the MD simulations, it appears the agitation enabled the naphthalene group of compound **31** to take place preferentially within the protein cavity overlay by Y82 and Y96 on both sides that allowed the hydroxypiperidinone group to make strong polar contacts with PPI4K residues (Fig. 4c) which obviously relaxed the system. The binding pose of **31** retrieved after the system has converged is characterized by polar contacts between the hydroxypiperidinone group and the backbone atoms of Y96, V97 and T100 (Fig. 4d). A fourth hydrogen bond interaction was found between the terminal carbonyl moiety and K48 of *P*PI4K. The distance between the centroids (within < 4.0 Å to each other) and their orientation depict the presence of π-π interactions between the aromatic rings of **31** and the phenyl rings of Y82 and Y96. Taken together, the MD simulations studies confirm the binding hypothesis of compound **31**, and by extension the other derivatives, toward *P*PI4K and presents **31** as a viable virtual hit deserving experimental testing and other further biological studies.

## Conclusion

The aim of the fragment-based workflow described in this study was to identify small molecule(s) that could become starting material(s) in the development of new *P*PI4K inhibitor(s) for malaria therapeutics. A homology model of *P*PI4K based on human PI4K template was built and a dataset of compound fragments curated from ZINC15 database was docked towards the ATP-binding site of the *P*PI4K model. Two scaffolds uncommon among known PI4K ligands were identified amidst the fragment virtual hits. When similarity and substructure search was conducted to explore ligand space of the hits, we found that only the derivatives of one of them (indo group) demonstrated enhanced binding affinity. Importantly, the best-scored derivative of the indo functionality has attractive physico-chemical features required for a drug-like candidate and that is a vital quality considering that current known PI4K inhibitors are faced with solubility problem. MD simulations confirmed the stable binding of the best-scored. Finally, analysis of binding pose revealed essential moieties of the compound involved in binding interaction with *P*PI4K active site residues and this information is valuable for chemical modification of the compound to optimize binding affinity. On the other hand, the identified ligand interaction in this study could be used to generate pharmacopheric model that could be employed as a query in virtual screening protocol.

## Data Availability

All data are available in the manuscript.

## References

[1] Onoabedje EA, Ibezim A, Okoro UC, Batra S (2020). Synthesis molecular docking, antiplasmodial and antioxidant activities of new sulfonamido-pepetide derivatives. Heliyon.

[2] Ugwu DI, Okoro UC, Ukoha PO, Okafor S, Ibezim A, Kumar NM (2017). Synthesis, characterization, molecular docking and in vitro antimalarial properties of new carboxamides bearing sulphonamide. Eur J Med Chem.

[3] Onoabedje EA, Ibezim A, Okoro UC, Batra S (2021). New sulphonamide pyrrolidine carboxamide derivatives: synthesis, molecular docking, antiplasmodial and antioxidant activities. PLoS ONE.

[4] WHO Media Center. Fact sheet on malaria. 2020. https://www.who.int/en/news-room/fact-sheets/detail/malaria. Accessed 4 April 2020.

[5] Ngo-Hanna J, Ntie-Kang F, Kaiser M, Brun R, Efange SMN (2014). 1-Aryl-1,2,3,4-tetrahydroisoquinolines as potential antimalarials: synthesis, in vitro antiplasmodial activity and in silico pharmacokinetics evaluation. RSC Adv.

[6] De Rycker M, Horn D, Aldridge B, Amewu RK, Barry CE, Buckner FS (2020). Setting our sights on infectious diseases. ACS Infect Dis.

[7] Uwimana A, Legrand E, Stokes BH, Ndikumana JM, Warsame M, Umulisa N (2020). Emergence and clonal expansion of in vitro artemisinin-resistant *Plasmodium falciparum* kelch13 R561H mutant parasites in Rwanda. Nat Med.

[8] Bousema T, Drakeley C (2011). Epidemiology and infectivity of *Plasmodium falciparum* and *Plasmodium vivax* gametocytes in relation to malaria control and elimination. Clin Microbiol Rev.

[9] McNamara CW, Lee MCS, Lim CS, Lim SH, Roland J, Nagle A (2013). Targeting *Plasmodium* PI(4)K to eliminate malaria. Nature.

[10] Paquet T, Le Manach C, Cabrera DG, Younis Y, Henrich PP, Abraham TS (2017). Antimalarial efficacy of MMV390048, an inhibitor of *Plasmodium* phosphatidylinositol 4-kinase. Sci Transl Med.

[11] Sternberg AR, Roepe PD (2020). Heterologous expression, purification, and functional analysis of the *Plasmodium falciparum* phosphatidylinositol 4-kinase IIIβ. Biochemistry.

[12] Fienberg S, Eyermann CJ, Arendse LB, Basarab GS, McPhail JA, Burke JE, Chibale K (2020). Structural basis for inhibitor potency and selectivity of *Plasmodium falciparum* phosphatidylinositol 4-kinase inhibitors. ACS Infect Dis.

[13] Paquet T, Manach CL, Cabrera DG, Younis Y, Henrich PP, Abraham TS, Lee MCS, Basak R, Ghidelli-Disse S, Lafuente-Monasterio MJ, Bantscheff M, Ruecker A, Blagborough AM, Zakutansky SE, Zeeman AM, White KL, Shackleford DM, Mannila J, Morizzi J, Scheurer S, Angulo-Barturen I, Martínez MS, Ferrer S, Sanz LM, Gamo FJ, Reader J, Botha M, Dechering KJ, Sauerwein RW, Tungtaeng A, Vanachayangkul P, Lim CS, Burrows J, Witty MJ, Marsh KC, Bodenreider C, Rochford R, Solapure SM, Jiménez-Díaz MB, Wittlin S, Charman SA, Donini C, Campo B, Birkholtz LM, Hanson KK, Drewes G, Kocken CHM, Delves MJ, Leroy D, Fidock DA, Waterson D, Street LJ, Chibale K (2017). Antimalarial efficacy of MMV390048, an inhibitor of *Plasmodium* phosphatidylinositol 4-kinase. Sci Transl Med.

[14] Fienberg S, Eyermann CJ, Arendse L, Basarab GS, McPhail J, Burke JE, Chibale K (2020). Structural basis for inhibitor potency and selectivity of *Plasmodium falciparum* phosphatidylinositol 4-kinase inhibitors. ACS Inf Dis.

[15] Ibrahim MAA, Abdelrahman AHM, Hassan AMA (2019). Identification of novel *Plasmodium falciparum* PI4KB inhibitors as potential anti-malarial drugs: homology modeling, molecular docking and molecular dynamics simulations. Comput Biol Chem.

[16] Younis Y, Douelle F, Feng TS, Cabrera DG, Manach CL, Nchinda AT (2012). 3,5-Diaryl-2-aminopyridines as a novel class of orally active antimalarials demonstrating single dose cure in mice and clinical candidate potential. J Med Chem.

[17] Reuberson J, Horsley H, Franklin RJ, Ford D, Neuss J, Brookings D, Huang Q, Vanderhoydonck B, Gao LJ, Jang MY, Herdewijn P, Ghawalkar A, Fallah-Arani F, Khan AR, Henshall J, Jairaj M, Malcolm S, Ward E, Shuttleworth L, Lin Y, Louat ST, Waer M, Herman J, Payne A, Ceska T, Doyle C, Pitt W, Calmiano M, Augustin M, Steinbacher S, Lammens A, Allen R (2018). Discovery of a potent, orally bioavailable PI4KIIIβ inhibitor (UCB9608) able to significantly prolong allogeneic organ engraftment in vivo. J Med Chem.

[18] Le-Manach C, Gonzàlez-Cabrera D, Douelle F, Nchinda AT, Younis Y, Taylor D (2014). Medicinal chemistry optimization of antiplasmodial imidazopyridazine hits from high throughput screening of a SoftFocus kinase library: part 1. J Med Chem.

[19] Ibezim A, Onuku RS, Ibezim A, Ntie-Kang F, Nwodo NJ, Adikwu MU (2021). Structure-based virtual screening and molecular dynamics simulation studies to discover new SARS-CoV-2 main protease inhibitors. Sci Afr.

[20] Kolb P, Kipouros CB, Huang D, Caflisch A (2008). Structure-based virtual screening and molecular dynamics simulation studies to discover new SARS-CoV-2 main protease inhibitors. Proteins.

[21] Kandepedu N, Gonzàlez-Cabrera D, Eedubilli S, Taylor D, Brunschwig C, Gibhard L (2018). Identification, characterization, and optimization of 2,8-disubstituted-1,5-naphthyridines as novel *Plasmodium falciparum* phosphatidylinositol-4-kinase inhibitors with *in vivo* efficacy in a humanized mouse model of malaria. J Med Chem.

[22] Kumar A, Voet A, Zhang KY (2012). Fragment based drug design: from experimental to computational approaches. Curr Med Chem.

[23] National Center for Biotechnology Information (NCBI). Bethesda (MD): National Library of Medicine (US), National Center for Biotechnology Information. 1988. https://www.ncbi.nlm.nih.gov/. Accessed 06 Apr 2021.

[24] Berman HM, Westbrook J, Feng Z, Gilliland G, Bhat TN, Weissig H, Shindyalov IN, Bourne PE (2000). The protein data bank. Nucleic Acids Res.

[25] Webb B, Sali A (2016). Comparative protein structure modeling using MODELLER. Curr Protoc Bioinform.

[26] Laskowski RA, MacArthur MW, Moss DS, Thornton JM (1993). PROCHECK: a program to check the stereochemical quality of protein structures. J Appl Crystallogr.

[27] Discovery Studio Visualizer Software, Version 4.0. 2019. http://www.accelrys.com. Accessed 10 May 2021

[28] Irwin JJ, Shoichet BK (2005). ZINC—a free database of commercially available compounds for virtual screening. J Chem Inf Model.

[29] Gaulton A, Bellis L, Chambers J, Davies M, Hersey A, Light Y, McGlinchey S, Akhtar R, Bento AP, Al-Lazikani B, Michalovich D, Overington JP (2012). ChEMBL: a large-scale bioactivity database for chemical biology and drug discovery. Nucleic Acids Res.

[30] O'Boyle NM, Banck M, James CA, Morley C, Vanderrmeersh T, Hutchison GR (2011). Open babel: an open chemical toolbox. J Cheminform.

[31] Ibezim A, Debnath B, Ntie-Kang F, Mbah CJ, Nwodo NJ (2017). Binding of anti-*Trypanosoma* natural products from African flora against selected drug targets: a docking study. Med Chem Res.

[32] Halgren TA (1996). Merck molecular forcefield. J Comput Chem.

[33] Chemical Computing Group. Molecular operating environment (MOE) software. 2014.

[34] Ibezim A, Onoabedje EA, Adaka IC, Omeje KO, Onoabedje US, Obi BC (2020). Carboxamides bearing sulfonamide functionality as potential novel phospholipase A2 inhibitors. ChemistrySelect.

[35] Jonathan BB, Georgian AH (2010). New substructure filters for removal of Pan Assay Interference Compounds (PAINS) from screening libraries and for their exclusion in bioassay. J Med Chem.

[36] Malde AK, Zuo L, Breeze M, Stroet M, Poger D, Nair PC, Oostenbrink C, Mark AE (2011). An automated force field topology builder (ATB) and repository: version 1.0. J Chem Theory Comput.

[37] Scott WRP, Hunenberger PH, Tironi IG, Mark AE, Bileter SR, Fennen J, Torda AE, Huber T, Kruger P, vanGunsteren WF (1999). The GROMOS biomolecular simulation program package. J Phys Chem.

[38] Kaczor AA, Silva AG, Loza MI, Kolb P, Castro M, Poso A (2016). Structure-based virtual screening for dopamine D2 receptor ligands as potential antipsychotics. ChemMedChem.

[39] Ibezim A, Nwodo NJ, Nnaji NJN, Ujam OT, Olubiyi OO, Mbah CJ (2016). In silico investigation of morpholines as novel class of trypanosomal triosephosphate isomerase inhibitors. Med Chem Res.

[40] Wang T, Wu M, Chen ZJ, Chen H, Lin JP, Yang LR (2015). Fragment based drug discovery and molecular docking in drug design. Curr Pharm Biotech.

[41] Qingxin L, Congbao K (2021). Perspecties on fragment-based drug discovery: a strategy applicable to diverse targets. Curr Topics Med Chem.

[42] Mejdrová I, Chalupská D, Placková P, Müller C, Sala M, Klíma M, Baumlová A, Hrebabecky H, Prochazkova E, Dejmek M, Strunin D, Weber J, Lee G, Matoušová M, Mertlíková-Kaiserová H, Ziebuhr J, Birkus G, Boura E, Nencka R (2016). Rational design of novel highly potent and selective phosphatidylinositol 4-kinase IIIβ (PI4KB) inhibitors as broadspectrum antiviral agents and tools for chemical biology. J Med Chem.

[43] Sengupta N, Jović M, Barnaeva E, Kim DW, Hu X, Southall N, Dejmek M, Mejdrova I, Nencka R, Baumlova A, Chalupska D, Boura E, Ferrer M, Marugan J, Balla T (2019). A large scale high-throughput screen identifies chemical inhibitors of phosphatidylinositol 4-kinase type II alpha. Methods.

[44] Ibezim A, Obi BC, Ofokansi NM, Mbah CJ, Nwodo NJ (2018). Discovery of trypanocidal bioactive leads by docking study, molecular dynamic simulation and in vivo screening. ChemistrySelect.

[45] Ibezim A, Nwodo NJ, Mbah CJ (2019). Computer aided drug design: an introduction. J Pharm Dev Ind Pharm.

